# Development and validation of the organizational health behavior index: a mixed-methods instrument for measuring organizational health

**DOI:** 10.3389/fpsyg.2025.1689559

**Published:** 2025-12-02

**Authors:** Abad Alzuman, Muath Al Jaafari, Zaiba Ali, Rahila Ali, Lina Ibrahim Bakadam

**Affiliations:** 1 Department of Management, College of Business Administration, Princess Nourah Bint Abdulrahman University, Riyadh, Saudi Arabia; 2 Organizational Culture and Internal Communication, ICM, Amjad Watan, Riyadh, Saudi Arabia; 3 Faculty of Management, Jagran Lakecity University, Bhopal, India

**Keywords:** organizational health, awareness, appreciation, relations, engagement, communication satisfaction, organizational culture, employee persona

## Abstract

**Background:**

Organizational health is crucial to promote employees’ well-being, sustainable performances, and long-term survival; existing tools are, however, frequently unidimensional and have been developed without consideration for the organizational context.

**Purpose:**

The purpose of this study was to construct and validate the Organizational Health Behavior Index (OHBI), a composite measure that includes quantitative and qualitative domains.

**Methods:**

A sequential mixed-methods design was used to survey 7,548 workers in various Saudi industries. Subscale A (quantitative) comprised awareness, appreciation, relations, engagement, and communication satisfaction, which were validated through EFA, CFA, and reliability. Subscale B (qualitative) included organizational culture, employee persona, and voice, which were analyzed for theme, interrater reliability, and expert triangulation.

**Results:**

CFA revealed a good fit (CFI = 0.960, TLI = 0.950, RMSEA = 0.053) with factor loadings > 0.70 and reliability *α* ≥ 0.707. Criterion-related validity demonstrated strong correlations (r = 0.75–0.83) with an existing model. The second aim we accomplished was to enhance our results by measuring employee-induced cultural perceptions (Subscale B).

**Conclusion:**

The OHBI is a reliable multidimensional measure of organizational health, providing both theoretical and practical value. Future validation work in other contexts is suggested.

## 1 Introduction

### 1.1 Conceptualizing organizational health

Organizational health refers to an organization’s capability to perform effectively, adapt to change, and maintain both employee well-being and long-term viability. Scholars have increasingly recognized it as a systemic attribute encompassing cultural, structural, and behavioral dimensions (Singh et al., 2017). A healthy organization is one in which people are committed, empowered, and proud to contribute to its goals (Lu et al., 2017). It fosters transparency, engagement, and resilience, enabling organizations to thrive in complex environments (Cahyadi et al., 2024). Apipalakul (2017) emphasized that organizational health represents the overall atmosphere of an organization and can be viewed as the opposite of corruption. By adhering to governing principles, regulations, and order, the administrative system functions effectively and contributes to organizational productivity. Similarly, Velarde et al. (2020) noted that the concept of organizational health has often been used in management literature as an abstract idea of a “good organizational structure.” They argued that a healthy environment not only enables organizations to survive within their environments but also equips them with the capacity to overcome difficulties and sustain long-term success.

Empirical studies further demonstrate that healthy organizations experience superior financial, operational, and human capital outcomes, such as increased employee engagement, reduced turnover, and stronger alignment between strategy and execution (Kuswati, 2020). A healthy organization is not built upon short-term goals; instead, it requires long-term strategy and execution. Its key pillars include organizational alignment, clear communication and information flow, employee well-being and development, fairness, purposeful work, and innovation (Nkansah-Dwamena, 2024).

Every organization should strive to promote organizational health because of its benefits. Regardless of whether it operates in the private or public sector, each organization aims to deliver high-quality services or outcomes to its target stakeholders (Merdrousians, 2024). Healthy organizations are better able to make sound decisions and deliver sustainable services. Just as health in living beings reflects the absence of disease or dysfunction, organizational health reflects the effective functioning of systems and structures (Amini and Sokhanvar, 2020). Comparable to serious diseases that can cause irreversible harm, structural or managerial deficiencies can undermine organizational performance (Shanafelt et al., 2019).

In response to the growing demand for comprehensive evaluation tools, governments, businesses, and academic institutions have developed various frameworks to assess organizational health. However, many existing models rely solely on either quantitative or qualitative methods. To address this gap, the *Organizational Health Behavior Index (OHBI)* was developed using a mixed-method approach that integrates both types of assessments. The instrument comprises two subscales: Subscale A (quantitative) and Subscale B (qualitative), enhancing its reliability, depth, and applicability across organizational contexts.

### 1.2 Organizational health behavior index: a revised and validated model

Despite the widespread use of organizational health frameworks such as Keller and Price’s (2011a,b) OHI, the WHO Healthy Workplace Model (World Health Organization [WHO], 2010), and culture/climate indices, most existing instruments remain limited to structural, policy, or cultural dimensions. These tools often overlook behavioral and relational indicators—such as appreciation, awareness, and communication satisfaction—that reflect employees’ lived experiences at work. Furthermore, the majority rely on either quantitative surveys or qualitative assessments in isolation, limiting their comprehensiveness. To address this limitation, the present study develops and validates the OHBI, which systematically integrates eight interrelated domains: awareness, appreciation, relations, engagement, internal communication satisfaction, culture, persona, and employee voice. By combining quantitative psychometric validation with qualitative assessments, the OHBI moves beyond existing tools by embedding both structural and relational dimensions of organizational health. The purpose of this study is therefore 2-fold: (1) to refine and validate the OHBI as a multidimensional instrument that captures organizational health through both observable behaviors and contextual factors and (2) to examine its psychometric properties across a large and diverse sample of employees in Saudi Arabia.

The aim of this study is to develop and validate an updated version of the OHBI. A multidimensional diagnostic tool that combines quantitative and qualitative dimensions to address gaps in existing models and support organizational health measurement in diverse contexts based on the previous study by Jaafari et al. (2023). The present study contributes to the literature in three significant ways. First, it advances theory by integrating the job demands–resources (JD-R) model, social exchange theory, and the competing values framework (CVF) to provide a multidimensional understanding of organizational health. Second, it strengthens methodological rigor by employing a comprehensive validation strategy that includes EFA, CFA, reliability testing, criterion validity, and qualitative triangulation. Third, it offers practical value by introducing an evidence-based tool tailored to the Saudi Vision 2030 context, enabling organizations to enhance employee engagement, communication, and culture for sustained organizational well-being. A key addition is the integration of the Employee Net Promoter Score (eNPS) as a sub-variable under the employee engagement construct, strengthening the instrument’s ability to capture advocacy behavior and organizational loyalty. Furthermore, the construct previously labeled as *internal communication* has been reconceptualized and relabeled as communication satisfaction, providing a more accurate measure of employees’ perceived quality and effectiveness of organizational communication processes.

## 2 A literature review

### 2.1 Conceptual foundations of organizational health

According to Miles, organizational health, which refers to sustaining life in its environment, constantly developing itself, coping with problems, and having the ability to live and develop these abilities, is regarded as an essential component of success in today’s business organizations. In particular, the idea of organizational health, which has begun to be employed by academics researching management, human resource management, and industrial psychology, has been the topic of much research (Vural, 2013). Organizational health refers to an organization’s ability to align with a shared vision and adapt to changes to achieve business objectives. It demonstrates a company’s ability to respond to shifting demands. Soehartono et al. (2023) defined organizational health as an organization’s ability to deal with various tensions, such as opposing ideals and dialectical views. It also facilitates an organization’s evolution and influences functionality (Doganay A. and Dagli, 2020; Doganay E. and Dagli, 2020). All companies must work as a cohesive unit to achieve a common goal; organizational health is attainable.

### 2.2 Measurement approaches to organizational health

The Organizational Health Index (OHI) developed (Keller and Price, 2011a,b), provides a robust framework for evaluating organizational vitality through three key dimensions: internal alignment, which ensures that an organization’s vision, strategy, culture, and climate are synchronized; execution quality, which reflects the organization’s effectiveness in service delivery; and renewal capacity, which assesses the ability to sense, adapt to, and influence the external environment. This model has been widely applied across sectors. A study by Telkom Indonesia (Harjanti and Gustomo, 2017) found that most OHI dimensions were rated at the “Elite” level, with the exception of *Innovation and Learning*. Telkom’s dominant organizational archetype was *Market Focus*, consistent with its role as a service-oriented company, followed by *Leadership Driven*, *Knowledge Core*, and *Execution Edge* archetypes. Doganay E. and Dagli (2020) proposed a framework tailored to educational institutions, comprising four dimensions: Academic Emphasis, which highlights the importance of teacher-student interactions and support resources; Morale, which captures peer relationships, organizational climate, and leadership influence; Supportive Leadership, reflecting participatory management, open communication, and respect for teacher involvement; and Environmental Factors, addressing non-academic aspects, such as school safety and discipline systems. Together, these measurement models demonstrate the multidimensional nature of organizational health and the need for frameworks that are both theoretically grounded and contextually adaptable.

While existing tools such as the Centers for Disease Control and Prevention (CDC) Worksite Health ScoreCard emphasize the presence of organizational health promotion policies and practices, they primarily evaluate structural interventions (e.g., policies and programs related to physical activity, nutrition, and chronic disease prevention) rather than behavioral or relational dimensions (Centers for Disease Control and Prevention, 2019; Glasgow et al., 2019). Similarly, organizational culture scales, such as the CVF, provide insights into value orientations and managerial styles but do not directly assess employees’ awareness, appreciation, or communication satisfaction as behavioral indicators of organizational health (Cameron and Quinn, 2011; Hartnell et al., 2011). The OHBI advances this literature by integrating both quantitative and qualitative measures that capture organizational-level health behaviors and employee perceptions. This multidimensional approach enables a richer diagnosis of organizational health, bridging the gap between structural assessments and cultural value frameworks. Therefore, the OHBI systematically incorporates both quantitative psychometric validation and qualitative narrative data, offering a holistic view of organizational health behaviors.

### 2.3 Crucial dimensions of organizations and their contribution to organizational health

#### 2.3.1 Awareness and organizational health

Awareness is a multidimensional construct that plays a vital role in an organization’s ability to anticipate, interpret, and respond to change (Hayajneh et al., 2021). According to Lowdermilk and Hammontree (2020), awareness enables decision-makers to assess both the internal and external environment, thereby enhancing their ability to respond proactively to emerging challenges. Strategic awareness, in particular, contributes to organizational readiness by helping leaders understand present conditions, anticipate potential risks, and allocate resources effectively to ensure the successful implementation of change initiatives.

Schein’s (1985) organizational culture model further emphasized the significance of awareness in navigating organizational dynamics. At its core, the model highlights *espoused values*—explicitly stated norms and policies—as central to aligning employee behavior. Employees who are aware of and resonate with these values are more likely to develop a sense of belonging and purpose, which in turn fosters stronger organizational commitment and engagement. In this sense, awareness functions not only as a strategic capability but also as a cultural mechanism. It equips organizations to remain adaptive and resilient, while simultaneously strengthening the psychological connection between employees and organizational goals (Akpa, 2021).

#### 2.3.2 Appreciation and organizational health

Appreciation, often framed as gratitude, is a core psychological resource that strengthens individual and organizational well-being. From a positive psychology lens, gratitude can be cultivated through training within a primary prevention framework, enhancing efficiency, productivity, and prosocial behaviors at work (Di Fabio et al., 2017). Fredrickson’s (2000) *Broaden-and-Build Theory* explained that positive emotions expand cognitive flexibility and build lasting personal and social resources, with ripple effects across teams and customer relations (Vacharkulksemsuk and Fredrickson, 2013). Similarly, the *Psychological Capital (PsyCap) model* (Luthans et al., 2007) embeds gratitude-related constructs such as hope and optimism, shown to improve resilience, performance, and satisfaction. Lin (2017) found that higher-order gratitude predicts better psychological well-being, while Luthans et al. (2017) confirmed gratitude’s role in boosting engagement and resilience. Thus, appreciation functions not only as an individual trait but also as a relational and organizational resource that fosters positive climates and sustainable organizational health.

#### 2.3.3 Relation and organizational health

A supportive work environment—characterized by empathy, encouragement, and positive feedback—enables employees to thrive, particularly in uncertain times like the COVID-19 pandemic (Colbert et al., 2016). Supportive peer relationships, including task assistance, emotional support, and knowledge sharing, foster resilience and are strongly associated with commitment, well-being, belonging, and engagement (Chen and Feeley, 2012). Organizations play a critical role in sustaining employee mental health by providing resources that reduce stress and strengthen resilience (Kelloway et al., 2012; Kizuki and Fujiwara, 2020). When employees feel valued and supported, they are more likely to develop trust, psychological safety, and long-term engagement (Wright and Huang, 2012). The JD-R model (Bakker and Demerouti, 2007) further explains that workplace resources—especially social support—not only buffer against stress but also enhance motivation, performance, and sustainable organizational health.

#### 2.3.4 Employee engagement and organizational health

Employee engagement, first conceptualized by Kahn (1990) as the physical, cognitive, and emotional investment of employees in their roles, reflects a sense of belonging that has evolved with changing work demands (Sun and Bunchapattanasakda, 2019). Strongly tied to organizational health, engagement is shaped by alignment, communication, well-being, fairness, and meaningful work (Karpenkova, 2022). Engaged employees not only perform better—92% of executives affirm its organizational benefits (Harvard Business Review, 2020)—but also contribute to sustainability and long-term performance (Leitão et al., 2019). Critically, engagement is both an outcome and a driver of organizational health. Frameworks such as Kahn’s psychological conditions, the JD-R model (Bakker and Demerouti, 2007), and Gallup’s Q12 (Harter et al., 2002) highlight how intentional design of supportive environments fosters productivity, retention, and resilience.

#### 2.3.5 Internal communication and organizational health

Effective communication is a strategic necessity for organizational survival and growth, as it directly supports employee success, performance, and engagement (Ali et al., 2021). Internal communication consists of multiple dimensions serving distinct purposes (Bowman, 2020) and plays a critical role in shaping employee attitudes, especially in times of uncertainty, such as the pandemic (Grates, 2020). Through vertical (top-down and bottom-up) and horizontal exchanges (Sollitto and Myers, 2015), organizations can foster belonging and resilience when communication is open, two-way, participatory, and genuinely attentive to employee needs (Qin and Men, 2023). Moreover, effective internal communication reinforces organizational culture by strengthening trust and shared values (Yue et al., 2021). Environments that combine guidance, support, and dialog between employees and leadership empower individuals to navigate challenges more effectively (Brown and Roloff, 2015).

#### 2.3.6 Organizational culture and organizational health

Organizational culture refers to shared assumptions, attitudes, and ideas that guide how employees behave, shaping their conduct, performance, and interactions within defined boundaries (Lubis et al., 2020). The CVF, developed by Quinn and Rohrbaugh (1981), is widely used to assess culture by categorizing it into four types: clan, adhocracy, market, and hierarchy (Cameron and Quinn, 2011). It rests on two dimensions: flexibility versus control, and internal versus external orientation, with the inherent tensions between these values giving the framework its name.

#### 2.3.7 Employee persona

An employee persona is a profile of a specific employee segment that details their characteristics, attitudes, wants, and needs (Masionis, 2024). HR teams can use these profiles to better personalize and tailor their initiatives to best suit their employees (Culture Amp, 2021). It is crucial for effective HR practices, employee engagement, and creating a positive workplace culture by allowing targeted interventions and communication based on specific employee needs (Robertson, 2024).

#### 2.3.8 Employee voice

Employee voice refers to individuals having the ability to express their viewpoints regarding their work safely and openly, regardless of the time, place, or method of communication. When employee voice mechanisms function effectively, individuals feel valued, trusted, and empowered, which in turn enhances their job satisfaction and overall performance. A workplace that actively encourages employee voice fosters stronger relationships between employers and employees, creating a more inclusive and engaging work environment. Conversely, when employees feel unheard, their well-being, commitment, and capacity for innovation may be adversely affected (Chartered Institute of Personnel and Development [CIPD], 2023). Creating a workplace culture where employees feel comfortable expressing their thoughts and ideas significantly enhances various aspects of organizational effectiveness, ultimately impacting overall performance. When employees feel that their voices are heard, engagement levels rise, with research indicating that 74% of individuals feel more connected to their work when they believe their input is valued (Broderick, 2024).

#### 2.3.9 Theoretical framework

The development of the OHBI is grounded in multiple theoretical perspectives that collectively explain the interaction between organizational structures, employee behaviors, and workplace outcomes. The JD-R model (Bakker and Demerouti, 2007) provides the foundation for linking organizational resources such as awareness, appreciation, and communication with employee engagement and well-being, emphasizing how resources buffer job demands and foster motivation. Social exchange theory (Blau, 1964) further explained the reciprocal relationship between organizations and employees, where supportive behaviors such as recognition and appreciation lead to greater commitment and trust. The dimension of communication satisfaction (Downs and Hazen, 1977) underpins the role of internal communication in shaping alignment, transparency, and trust, which are essential for organizational health. In addition, the CVF (Cameron and Quinn, 2011) situates organizational culture within four competing orientations—clan, adhocracy, market, and hierarchy—helping to explain how cultural environments shape awareness, relations, and engagement.

**Figure 1:**
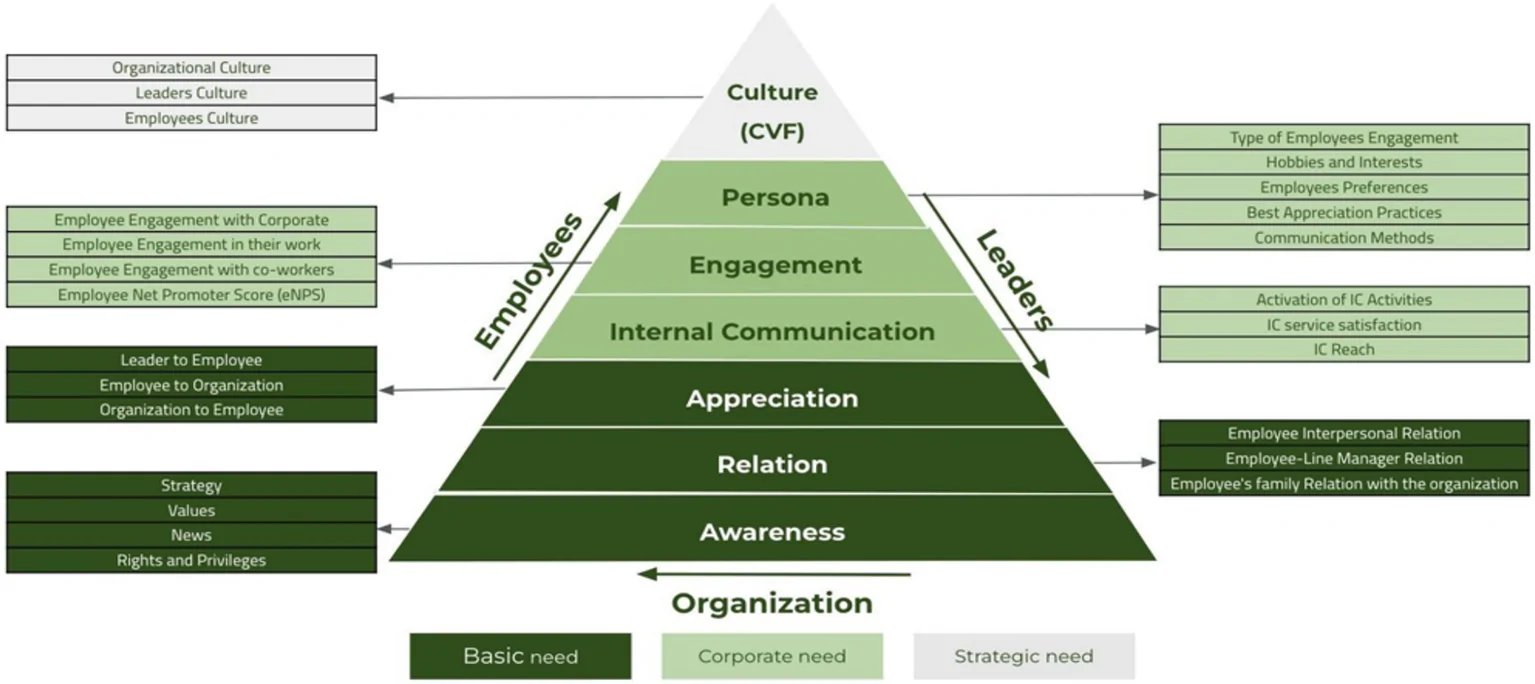
OHBI framework. The framework integrates eight interrelated dimensions—Awareness, Relations, Appreciation, Internal Communication, Engagement, Persona, Culture, and Voice—into a multilevel structure connecting organizational needs (basic, corporate, and strategic) with employee and leadership practices.

Finally, emerging HR approaches such as employee persona (Culture Amp, 2021; Robertson, 2024) and employee voice (Chartered Institute of Personnel and Development [CIPD], 2023) expand the framework by emphasizing personalization and participatory practices, reflecting the lived experiences of employees within organizations. Together, these theoretical perspectives provide the conceptual foundation for OHBI, enabling it to capture not only structural and cultural aspects of organizational health but also the behavioral and relational dimensions that drive sustainable engagement (Figure 1).

*Hypothesis 1 (H1)*: The Organizational Health Behavior Index is determined by several latent factors of the measure.

## 3 Methods

### 3.1 General overview of methodology

This study on the OHBI employs a mixed-method approach, comprising a quantitative subscale (Subscale A) and a qualitative subscale (Subscale B). Consequently, the validity and reliability of each subscale are assessed independently through a systematic and rigorous methodology. The validation process is structured across multiple phases and sample groups, integrating both quantitative and qualitative research strategies to ensure the robustness and reliability of the measurement instrument.

For the quantitative analyses, exploratory factor analysis (EFA) and reliability testing were conducted using IBM SPSS V 25 Statistics, while confirmatory factor analysis (CFA) and structural equation modeling (SEM) were performed using IBM AMOS v 20. The qualitative coding and interrater reliability (Cohen’s Kappa) were analyzed using SPSS v 25 (Table 1).

**Table 1:** Summary of phases, samples, and methods for OHBI development and validation.

Subscale	Phase	Study	Sample/experts	Objective	Methods used
Subscale A (Quantitative)	Phase 1	Study 1	8 Subject Experts	Item generation and content validity	Expert evaluation of clarity, relevance, representativeness
		Study 2	*n* = 1,066	Factor structure exploration	Exploratory Factor Analysis (EFA) using PAF and Promax rotation
	Phase 2	Study 3	*n* = 7,548	Internal consistency	Cronbach’s Alpha, Composite Reliability (CR)
		Study 4	*n* = 7,548	Construct validation	Confirmatory Factor Analysis (CFA), Convergent (AVE), Discriminant (HTMT, FL)
	Phase 3	Study 5	*n* = 244	Criterion-related validity	Pearson’s correlation with OH Model (OACA); *r* = 0.75–0.83
Subscale B (Qualitative)	Phase 4	Study 6	5 Subject Experts	Item generation and content validation	Thematic review based on CVF, SIT, and employee voice theories
	Phase 5	Study 7	*n* = 50	Face validity and cognitive pretesting	Pilot testing across diverse sectors
		Study 8	3 Expert Raters	Interrater reliability for qualitative coding	Thematic analysis, Cohen’s Kappa = 0.84

#### 3.1.1 Subscale A: quantitative validation

The quantitative component consisted of two key phases: item development and psychometric validation.

Phase 1 (Studies 1–2): Item generation was guided by an extensive literature review and benchmarking of existing organizational health and engagement instruments. Eight subject-matter experts assessed the initial item pool for content validity, ensuring relevance, clarity, and coverage of the constructs. Subsequently, EFA was conducted using data from 1,066 respondents, satisfying the recommended minimum subject-to-item ratio of 10:1 (Hair et al., 2018; Costello and Osborne, 2005). Phase 2 (Studies 3–4): Reliability and construct validity were examined using a larger sample (*n* = 7,548), enabling robust analysis. Internal consistency was assessed using Cronbach’s alpha and composite reliability (CR), with thresholds ≥ 0.70 indicating acceptable reliability (Nunnally and Bernstein, 1994; Fornell and Larcker, 1981). CFA was used to validate the factor structure. Convergent validity was assessed via average variance extracted (AVE ≥ 0.50) and CR, while discriminant validity was evaluated using the Fornell–Larcker criterion and the heterotrait–monotrait ratio of correlations (HTMT) (Henseler et al., 2015).

#### 3.1.2 Subscale B: qualitative validation

The qualitative dimension of the OHBI was developed and validated through two sequential phases. Phase 3 (Study 5): Items for the qualitative subscale were generated through consultations with five domain experts in human resource management and organizational culture. These experts evaluated the conceptual coverage, thematic clarity, and relevance of each construct, ensuring strong content validity (Creswell and Plano Clark, 2018).

Phase 4 (Studies 6–7): A qualitative content analysis was conducted on open-ended responses from 50 participants to identify recurring themes, patterns, and context-specific insights. Interrater reliability was assessed using ratings from three independent expert coders, producing a high level of agreement and affirming the credibility, dependability, and rigor of the qualitative coding process (Miles et al., 2014).

### 3.2 Research design and rationale

This study adopted a sequential mixed-methods research design, combining both quantitative and qualitative approaches to provide a comprehensive validation of the OHBI. The choice of a mixed-methods design was grounded in its ability to integrate numerical rigor with contextual insights, offering both psychometric precision and practical interpretation (Creswell and Plano Clark, 2018). Unlike purely quantitative methods, which may overlook nuanced perceptions of employees, or purely qualitative approaches, which may lack statistical generalizability, this design allows for a holistic exploration of organizational health by merging statistical validation with narrative understanding. Quantitative methods (EFA, CFA, reliability, and criterion validity) were selected to ensure construct accuracy and generalizability across large samples, while qualitative triangulation (Subscale B) was used to capture deeper insights into organizational culture, employee persona, and voice. By incorporating both paradigms, this study achieves greater explanatory depth and enhances the ecological validity of the OHBI.

## 4 Results

### 4.1 Subscale A: quantitative scale validation

#### 4.1.1 Phase 1

##### 4.1.1.1 Study 1: item generation for Subscale A (quantitative)

The development of the OHBI in this study builds upon a previously validated version of the scale. Subscale A (quantitative) was designed to capture key dimensions of organizational health, including awareness, appreciation, communication satisfaction, employee engagement, and relation. For the current validation, the item pool was primarily retained from the earlier version of the OHBI; however, two significant modifications were introduced to enhance construct coverage and measurement precision. First, the employee engagement subscale was updated to include a new sub-variable: eNPS, which captures employees’ likelihood to recommend their organization—an increasingly recognized proxy for affective commitment and engagement in contemporary organizational diagnostics (Oluwatobi et al., 2024). Second, the internal communication construct was reframed and reworded as communication satisfaction, reflecting evolving theoretical distinctions and aligning more closely with respondent feedback from earlier applications. The item language was revised to improve clarity and contextual relevance.

To establish content validity, the item pool was evaluated by eight expert raters with academic and professional expertise in organizational behavior, scale development, and psychometrics. Experts assessed each item for clarity, relevance, representativeness, and dimensional alignment using a structured evaluation matrix. Based on their feedback and consensus ratings, all items were retained. This expert validation process followed standard guidelines in psychometric development (DeVellis, 2016; Boateng et al., 2018), ensuring that the retained items adequately represented the theoretical domains of organizational health behavior.

###### 4.1.1.1.1 Sample size determination

The number of respondents was determined based on established psychometric guidelines for scale development and validation. For EFA, we followed the recommended subject-to-item ratio of at least 10:1 (Hair et al., 2019a,b; Costello and Osborne, 2005), which required a minimum of 170 respondents for the 17-item OHBI. We exceeded this benchmark by recruiting 1,066 participants for EFA to ensure stable factor extraction. For CFA, a much larger independent sample of 7,548 employees was employed to enhance generalizability and model robustness, consistent with best practices in SEM (Byrne, 2010; Brown, 2015). Finally, for criterion-related validity, a separate subsample of 244 professionals from a different sector was used to assess external convergence with the OACA model. This multi-sample strategy ensured both methodological rigor and external validity of the OHBI scale.

##### 4.1.1.2 Study 2: exploratory factor analysis

###### 4.1.1.2.1 Participants description

For Study 2, a total of 1,119 participants employed in the information technology sector in the Kingdom of Saudi Arabia were recruited using a convenience sampling method via Google Forms. Sample sizes exceeding 200 are considered adequate for most factor analyses (Tabachnick and Fidell, 2019). After screening for availability and willingness to participate, 1,066 responses were retained, with 767 identifying as male and 299 as female. Approximately 70% (*n* = 746) of the participants were between the ages of 26 and 36, with an average age of 31.5 years. One of the inclusion criteria was at least 24 months of experience in the current organization.

###### 4.1.1.2.2 Preliminary analysis

Descriptive statistics for all 17 items are presented in Table 2. The means ranged from 3.10 (AW4) to 4.15 (AW2), indicating moderate to high agreement among respondents. The skewness and kurtosis values for all items fall within the acceptable range of ±1.5, suggesting approximate normality (Demir, 2022; Hair et al., 2010; Byrne, 2010; Tabachnick and Fidell, 2013). Thus, the dataset was deemed suitable for factor analysis.

**Table 2:** Descriptive statistics.

Item	Mean	Std. deviation	Skewness	Std. error (Skew)	Kurtosis	Std. Error (Kurtosis)
AW1	3.76	1.150	−0.699	0.075	−0.351	0.150
AW2	4.15	0.958	−1.156	0.075	1.050	0.150
AW3	3.64	1.089	−0.477	0.075	−0.480	0.150
AW4	3.10	1.290	−0.067	0.075	−1.055	0.150
AP1	3.86	1.091	−0.837	0.075	0.045	0.150
AP2	3.76	1.288	−0.750	0.075	−0.580	0.150
AP3	3.46	1.189	−0.357	0.075	−0.755	0.150
RL1	3.84	1.138	−0.904	0.075	0.104	0.150
RL2	3.75	1.312	−0.744	0.075	−0.638	0.150
RL3	3.48	1.255	−0.458	0.075	−0.805	0.150
EE1	3.81	1.125	−0.718	0.075	−0.329	0.150
EE2	3.64	1.232	−0.636	0.075	−0.507	0.150
EE3	3.59	1.210	−0.466	0.075	−0.746	0.150
EE4	3.45	1.266	−0.587	0.075	−0.319	0.150
CS1	3.77	1.158	−0.672	0.075	−0.431	0.150
CS2	3.74	1.129	−0.551	0.075	−0.584	0.150
CS3	3.75	1.077	−0.583	0.075	−0.344	0.150

###### 4.1.1.2.3 Sampling adequacy and factorability

The Kaiser–Meyer–Olkin (KMO) measure of sampling adequacy was 0.807, exceeding the minimum acceptable threshold of 0.60 (Kaiser, 1970), while Bartlett’s test of sphericity was significant (*χ^2^* = 5040.049, df = 136, *p* < 0.001), confirming sufficient inter-item correlations for factor analysis (Bartlett, 1954). These results, summarized in Table 3, support the suitability of the dataset for EFA.

**Table 3:** Presents KMO and Bartlett’s test of sampling adequacy to justify the use of EFA.

Test	Value
Kaiser–Meyer–Olkin (KMO) measure of sampling adequacy	0.807
Bartlett’s test of sphericity	
Approx. Chi-Square	5040.049
Degrees of freedom (df)	136
Significance (*p*-value)	< 0.001

###### 4.1.1.2.4 Factor extraction and structure

**Figure 2:**
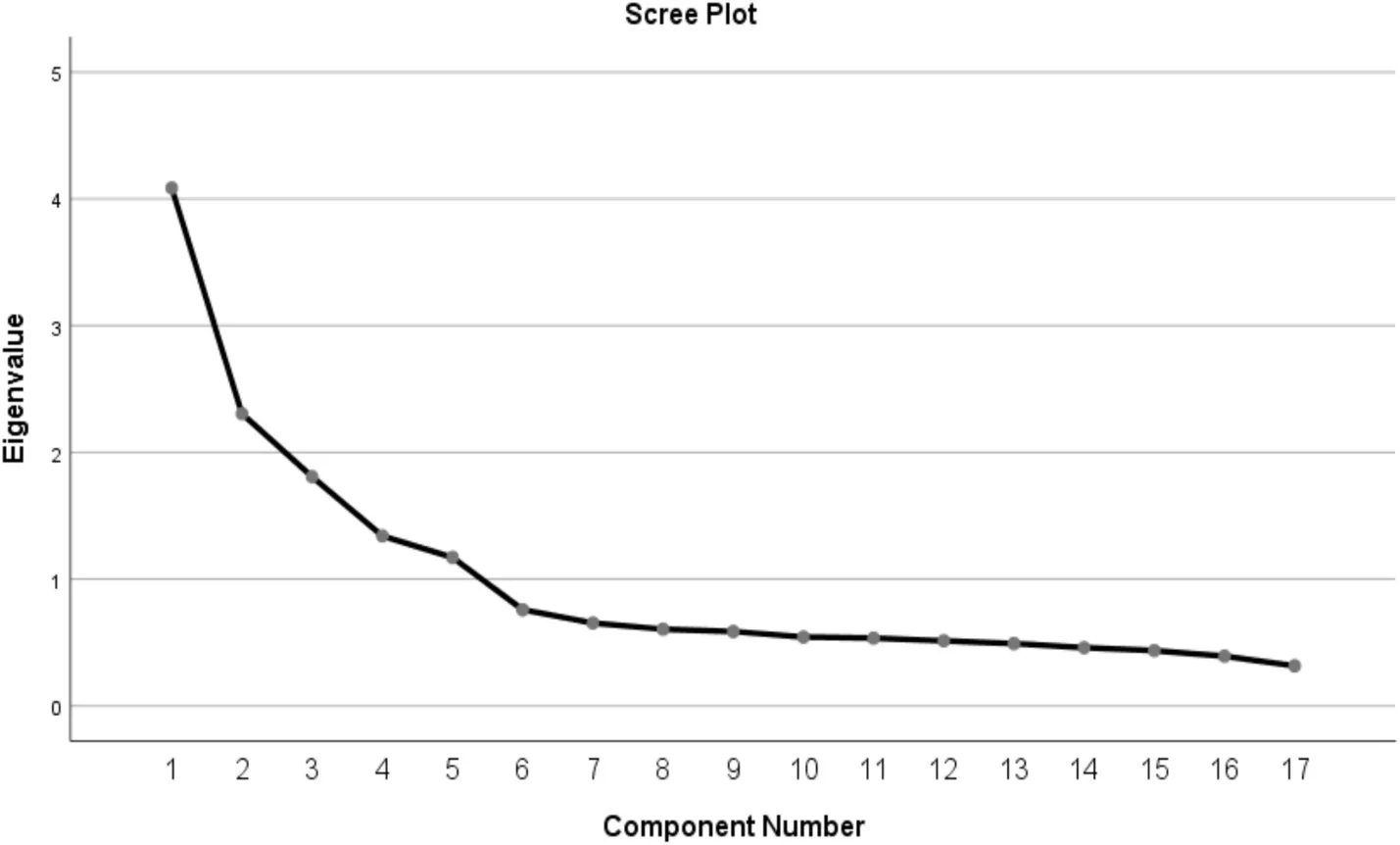
The scree plot shows five factors with eigenvalues much higher than 1, the cutoff value.

An EFA was conducted using principal axis factoring (PAF) with Promax rotation, given the assumption of correlated factors. The eigenvalues > 1 criterion and scree plot guided factor retention decisions (Hair et al., 2010; Costello and Osborne, 2005). As shown in Table 4 and Figure 2, five factors with eigenvalues >1 were extracted, cumulatively accounting for 63.54% of the total variance.

**Table 4:** Displays total variance explained using principal axis factoring (*n* = 1,066) extraction method: principal axis factoring.

Factor	Initial eigenvalues			Extraction sums of squared loadings			Rotation sums of squared loadingsa
	Total	% of Variance	Cumulative %	Total	% of Variance	Cumulative %	Total
1	5.801	34.125	34.125	5.435	31.972	31.972	3.325
2	2.358	13.873	47.999	2.008	11.814	43.786	4.323
3	2.180	12.822	60.821	1.828	10.755	54.541	3.635
4	1.234	7.256	68.077	0.897	5.275	59.816	2.119
5	1.004	5.905	73.982	0.632	3.720	63.536	4.014
6	0.504	2.965	76.948				
7	0.458	2.693	79.641				
8	0.409	2.408	82.049				
9	0.401	2.359	84.407				
10	0.394	2.316	86.723				
11	0.380	2.237	88.960				
12	0.365	2.146	91.106				
13	0.351	2.065	93.171				
14	0.334	1.967	95.138				
15	0.308	1.811	96.949				
16	0.275	1.619	98.568				
17	0.244	1.432	100.000				

Extraction method: Principal Axis Factoring was used for extraction. Rotation method: Oblimin with Kaiser Normalization (Promax). An eigenvalue of “a” would typically mean that the factor explains “a” amount of variance in the observed variables.

###### 4.1.1.2.5 Factor loadings and interpretability

The rotated factor structure presented in Table 5 revealed a coherent clustering of items into five distinct and interpretable dimensions: awareness (AW1–AW4), employee engagement (EE1–EE4), communication satisfaction (CS1–CS3), relations (RL1–RL3), and appreciation (AP1–AP3). All items demonstrated strong primary loadings (≥0.68) on their intended factors, reflecting high internal consistency and clear construct representation. Importantly, no items exhibited problematic cross-loadings or low factor loadings (<0.50), thereby confirming the psychometric robustness of the scale. These results align with established best practices in EFA (Velicer and Jackson, 1990; Costello and Osborne, 2005) and provide empirical support for the multidimensional structure of the OHBI. Collectively, this factor solution reinforces the theoretical underpinnings of the OHBI and its utility in capturing diverse yet interrelated aspects of organizational health-related behaviors.

**Table 5:** Components matrix presents factor loadings using Principal Axis Factoring.

Items	Component 1 (awareness)	Component 2 (engagement)	Component 3 (communication satisfaction)	Component 4 (relation)	Component 5 (appreciation)
AW1	0.804				
AW2	0.762				
AW3	0.771				
AW4	0.688				
EE1		0.696			
EE2		0.759			
EE3		0.809			
EE4		0.692			
CS1			0.814		
CS2			0.885		
CS3			0.857		
RL1				0.796	
RL2				0.807	
RL3				0.813	
AP1					0.803
AP2					0.807
AP3					0.749

###### 4.1.1.2.6 Scree plot analysis

The scree plot (see Figure 2) illustrates a distinct “elbow” at the fifth factor, supporting the five-factor solution. This graphical method, together with Kaiser’s criterion, further validated the dimensionality of the OHBI construct (Table 6).

**Table 6:** Cronbach’s alpha for 17-item OHBI scale.

Reliability statistics		
Cronbach’s alpha	Cronbach’s alpha based on standardized items	*N* of items
0.885	0.928	17

#### 4.1.2 Phase 2

##### 4.1.2.1 Study 3: internal consistency for quantitative scale (Subscale A)

Table 7 presents the reliability and validity statistics of the OHBI dimensions. Cronbach’s alpha values (0.885–0.921) confirm strong internal consistency. In addition, Dijkstra–Henseler’s Rho_A (~0.86–0.90), CR (0.838–0.879), and AVE (0.581–0.661) all meet recommended thresholds (Hair et al., 2019a,b). These results provide robust evidence of convergent validity and internal consistency reliability across the five OHBI factors. Therefore, the factor-wise reliability coefficients suggested that the overall scale had a significantly high Cronbach’s alpha. Moreover, Cronbach’s alpha criteria retained five factors with 17 items of the OHBI for CFA. Hence, it supported the EFA results that suggested five factors can be considered.

**Table 7:** Factor-wise Cronbach’s alpha for OHBI scale.

Factor	No. of items	Cronbach’s α	Rho_A*	Rho_C (CR)	AVE
Overall OHBI scale	17	0.885	—	—	—
Awareness	4	0.909	~0.88*	0.847	0.581
Appreciation	3	0.897	~0.87*	0.838	0.633
Relations	3	0.900	~0.86*	0.841	0.638
Employee engagement	4	0.890	~0.89*	0.879	0.646
Communication satisfaction	3	0.921	~0.90*	0.854	0.661

* denotes an approximate (rounded) value.

##### 4.1.2.2 Reliability and validity

The internal consistency for all five OHBI subscales was high (see Table 7), which corroborates the validity of the measurement model. The Cronbach’s alpha values between 0.885 and 0.921 indicate that the scale items are highly correlated and measure latent constructs consistently. The CR values vary from 0.838 to 0.879 and are above the threshold of 0.70, indicating the constructs are stable. Furthermore, the AVE value of (0.581–0.661) also supports the convergent validity as more than 50% of the variance of each construct is explained by its indicators. This means that awareness, appreciation, relations, engagement, and communication satisfaction items possess a large amount of shared variance, and they are empirically separate but theoretically linked aspects of organizational health behavior.

Corrected item-total correlations (0.60 to 0.85) were analyzed to determine item performance, and all were above the recommended threshold of 0.50. This indicates that none of the items make a significant contribution toward the overall factor or scale. In the “Cronbach’s alpha if Item Deleted” column, shown slight (≤0.002 difference), suggesting that deleting one item would not increase the reliability of the scale—evidence of model internal robustness. These findings as a whole suggest that the OHBI-17 scale is a psychometrically sound measure with good internal consistency and theoretical integrity.

##### 4.1.2.3 Validity analysis

About validity, the CFA results (*χ*^2^ = 1517.18, CFI = 0.978, TLI = 0.969, RMSEA = 0.041) evidence adequate construct validity and model fit. The standardized factor loading (0.68 ~ 0.87) indicates that all indicators have a high loading on the latent construct, which means the items are reliable. The convergent validity analysis, as indicated by AVE and CR values, shows that items in a given construct are highly correlated, whereas the discriminant validity assessments (Fornell–Larcker criterion and HTMT ratios < 0) (Table 8).

**Table 8:** Corrected item-total statistics on an overall scale.

Items	Corrected item-total correlation	Cronbach’s alpha if item deleted
AW1	0.770	0.874
AW2	0.813	0.874
AW3	0.738	0.875
AW4	0.724	0.874
AP1	0.777	0.874
AP2	0.787	0.874
AP3	0.726	0.874
RL1	0.765	0.874
RL2	0.835	0.874
RL3	0.744	0.874
EE1	0.609	0.877
EE2	0.767	0.874
EE3	0.719	0.874
EE4	0.854	0.873
CS1	0.788	0.874
CS2	0.772	0.874
CS3	0.800	0.873

##### 4.1.2.4 Participants characteristics

The demographic profile of participants in summarized in Table 9, where it can be seen majority of the respondents were male (85.2%), with females representing 14.8% of the data collected. Employees were majorly based in Riyadh (68.9%), followed by Mecca (12.5%) and other provinces with smaller proportions, ensuring geographical diversity. Among the participants, most worked in the government sector (64.6%), with notable representation from the private (24.3%) and semi-government (11.1%) sectors. Key industries included airlines (25.7%), healthcare (21.5%), and funds/finance (15.2%). The majority of employees had over 4 years of tenure (78.2%), and the largest role category was specialists (67.2%), ensuring perspectives from experienced employees across diverse organizational contexts.

**Table 9:** Participants characteristics.

Variable	Category	*f*	%
Gender	Female	1,118	14.8
	Male	6,430	85.2
Location	Riyadh	5,200	68.9
	Al-Bahah	55	0.7
	Al-Jawf	112	1.5
	Al-Qassim	160	2.1
	Asir	68	0.9
	Eastern	349	4.6
	Hail	77	1.0
	Jazan	99	1.3
	Mecca	940	12.5
	Medina	71	0.9
	Northern Borders	233	3.1
	Tabuk	97	1.3
	Najran	87	1.2
Industry	Agriculture	96	1.3
	Airline	1942	25.7
	Art and Culture	135	1.8
	Construction	330	4.4
	Event Management	25	0.3
	F&B	47	0.6
	Finance	126	1.7
	Funds	1,148	15.2
	Government Affairs	305	4.0
	Health	1,623	21.5
	Insurance	106	1.4
	IPO	155	2.1
	Military	440	5.8
	Oil & Gas	201	2.7
	Real Estate	344	4.6
	Tech	10	0.1
	Telecom	515	6.8
Industry Type	Private	1834	24.3
	Government	4,876	64.6
	Semi Government	838	11.1
Tenure	More than 48 months	5,906	78.2
	24–48 months	1,131	15.0
	12–24 months	96	1.3
	6–12 months	232	3.1
	3–6 months	118	1.6
	Less than 3 months	65	0.9
Position	Advisor	157	2.1
	Manager	886	11.7
	Director	738	9.8
	Executive	152	2.0
	Specialist	5,072	67.2
	Team Lead	543	7.2

##### 4.1.2.5 Study 4: construct validation

###### 4.1.2.5.1 Confirmatory factor analysis

A study was conducted on a sample of 7,548 employees to run CFA, testing the five factors with 17 items developed from EFA. The goodness-of-fit model indices were found to be excellent (*χ*^2^ = 2418.198, *χ*^2^/df = 22.19, Normed Fit Index (NFI) = 0.958, RFI – Relative Fit Index = 0.948, GFI – Goodness of Fit Index = 0.964, IFI – Incremental Fit Index = 0.960, TLI – Tucker–Lewis Index (also known as the Non-Normed Fit Index, NNFI) = 0.950, RMSEA – Root Mean Square Error of Approximation = 0.053, and CFI – Comparative Fit Index = 960) (Table 10). The findings explained that all the model fit indices supported a good model fit. The results indicate that the OHBI-17 model provides a strong fit to the data.

**Table 10:** Confirmatory factor analysis fit indices for the eight-factor model (OHBI-17) (*n* = 7,548).

Model	*χ* ^2^	*χ*^2^/df	Df	NFI	RFI	IFI	TLI	CFI	RMSEA
Five-Factors Model	1517.18	13.9	109	0.977	0.967	0.978	0.969	978	0.041

**Figure 3:**
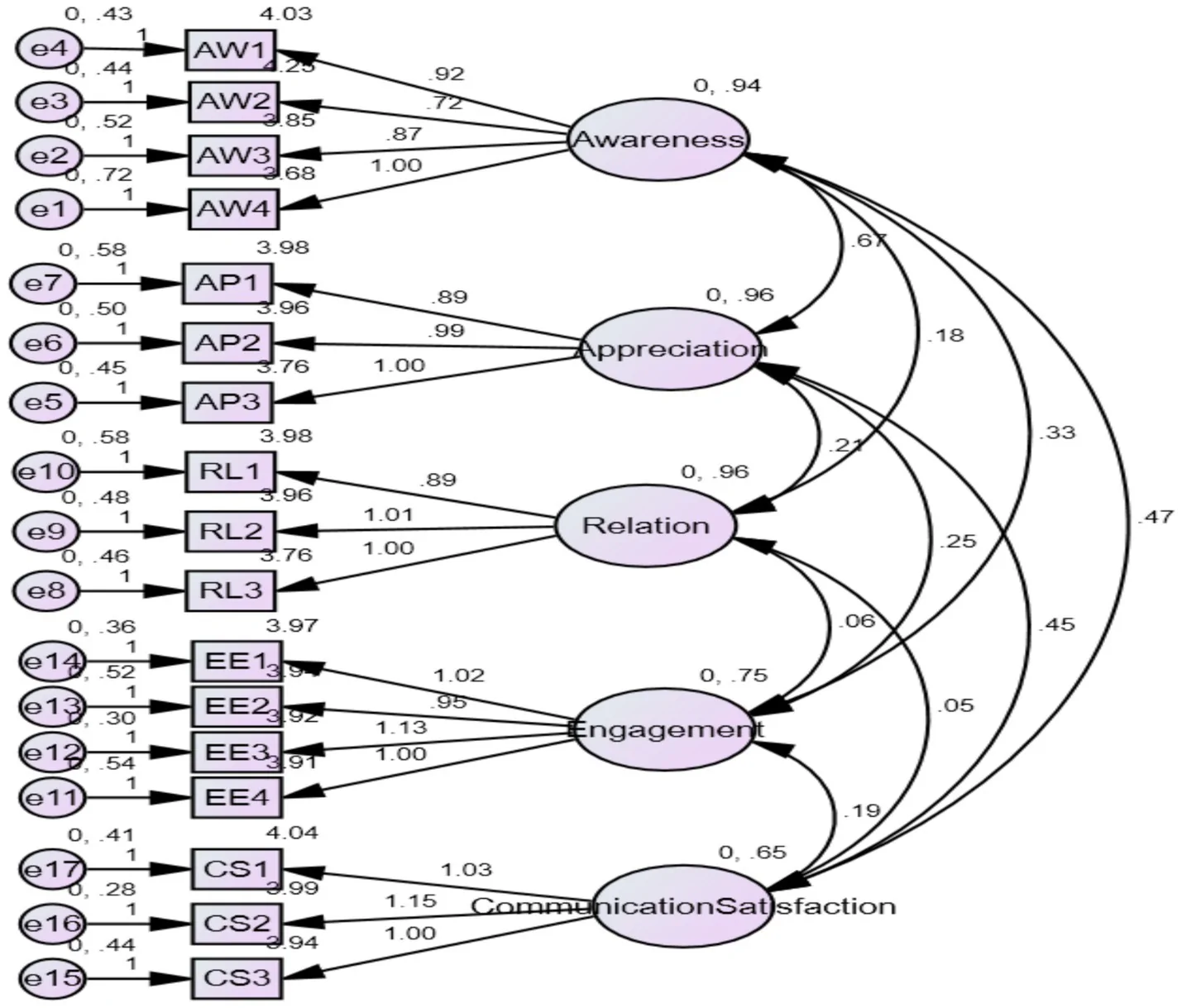
Structural path model of the organizational health behavior index (OHBI).

It can be concluded from the factor analysis that the final version of 17 items represents the five-factor measure of the OHBI (Figure 3 and Table 11). The standardized regression weights suggest that all constructs exhibit acceptable to strong factor loadings, with most values exceeding the recommended threshold of 0.7 (Hair et al., 2019a,b). The high values indicate that the measurement items adequately represent their respective constructs, supporting the reliability and validity of the model. The standardized factor loadings for the latent constructs awareness, appreciation, relation, engagement, and communication satisfaction indicate strong associations between the observed indicators and their respective constructs, with all values exceeding 0.70 and at *p*-values < 0.001 level of significance. This confirms the reliability and validity of the measurement model. Engagement (EE3 = 0.872) and communication satisfaction (CS2 = 0.871) show the highest factor loadings, suggesting these indicators are the most significant measures of their respective constructs. Similarly, appreciation (AP2 = 0.833) and relation (RL2 = 0.826) demonstrate strong representational power. The results validate the robustness of the model, indicating that the selected items effectively capture the underlying organizational constructs.

**Table 11:** Factor loadings/Standardized beta coefficients on five subscales of 17 items OHBI.

Factor	Item	Standardized factor loading
Factor 1: Awareness	(AW1)	0.807
	(AW2)	0.728
	(AW3)	0.761
	(AW4)	0.752
Factor 2: Appreciation	(AP1)	0.753
	(AP2)	0.808
	(AP3)	0.824
Factor 3: Relations	(RL1)	0.755
	(RL2)	0.819
	(RL3)	0.821
Factor 4: Employee engagement	(EE1)	0.827
	(EE2)	0.748
	(EE3)	0.871
	(EE4)	0.762
Factor 5: Communication satisfaction	(CS1)	0.792
	(CS2)	0.871
	(CS3)	0.773

The results confirmed the study’s hypothesis (H1), indicating that the Organizational Health Behavior Index is determined by multiple underlying latent factors of the measure.

###### 4.1.2.5.2 Convergent and discriminant validity

Convergent and discriminant validity assessments were used to examine the OHBI’s concept validity. Table 12 shows the AVE and the CR for each of the five components. All AVE values exceeded the required minimal level of 0.50 (Fornell and Larcker, 1981), suggesting good convergent validity. The constructs awareness (AVE = 0.565, CR = 0.842), appreciation (AVE = 0.621, CR = 0.830), relations (AVE = 0.647, CR = 0.845), employee engagement (AVE = 0.622, CR = 0.869), and communication satisfaction (AVE = 0.663, CR = 0.856) all showed strong internal consistency, with CR values exceeding 0.80, confirming indicator reliability and convergence.

**Table 12:** Convergent validity (AVE).

Construct	AVE	CR
Awareness	0.565	0.842
Appreciation	0.621	0.830
Relations	0.647	0.845
Employee engagement	0.622	0.869
Comm. satisfaction	0.663	0.856

Discriminant validity was determined using both the Fornell-Larcker criterion (Table 13) and the Heterotrait-Monotrait (HTMT) ratio (Table 14). According to the Fornell-Larcker technique, the square root of AVE for each construct (diagonal values) was greater than the corresponding inter-construct correlations, indicating adequate discriminant validity. Furthermore, HTMT values ranged from 0.085 to 0.820, falling considerably short of the conservative cut-off value of 0.85 (Henseler et al., 2015). These findings confirm that the constructs are conceptually unique and that the OHBI has strong psychometric qualities for both convergent and discriminant validity, making it appropriate for future SEM study.

**Table 13:** Discriminant validity (Fornell–Larcker criterion).

Construct	Awareness	Appreciation	Relations	Engagement	Communication satisfaction
Awareness	**0.752**				
Appreciation	0.704	**0.788**			
Relations	0.185	0.216	**0.804**		
Engagement	0.389	0.297	0.076	**0.788**	
Communication satisfaction	0.594	0.566	0.065	0.278	**0.814**

Bold values along the diagonal represent the square root of the Average Variance Extracted (√AVE), which are greater than the corresponding inter-construct correlations, thereby confirming discriminant validity (Fornell and Larcker, 1981).

**Table 14:** Discriminant validity (HTMT ratio).

Construct	Awareness	Appreciation	Relations	Engagement	Communication satisfaction
Awareness	–	0.820	0.228	0.455	0.645
Appreciation		–	0.250	0.350	0.605
Relations			–	0.095	0.085
Engagement				–	0.310
Comm. Satisfaction					–

#### 4.1.3 Phase 3

##### 4.1.3.1 Study 5: validity of Subscale A (quantitative scale)

Study 5 was conducted on a third sample comprising 244 professionals (110 males and 134 females) working in various educational institutions in Riyadh, Saudi Arabia, to establish the criterion validity of the OHBI. Among the participants, 84 were Saudi nationals and 160 were expatriates. To validate the OHBI, it was correlated with the Brief Questionnaire on Organizational Health Model used in the Aviation Organization of Tunisia (OACA). While existing Organizational Health models, such as the OACA, provide valuable insights, they lack an integrated approach that combines both quantitative and qualitative measures. Recognizing this limitation, the current study aimed to refine and validate the OHBI as a comprehensive, multidimensional diagnostic tool, incorporating modifications to its factors and testing it on a large and diverse population within the Kingdom of Saudi Arabia. The OHBI integrates both qualitative narratives and quantitative assessments, offering a more holistic view of organizational health. To establish criterion-related validity, OHBI results were compared against the established OACA model, thereby ensuring the new index accurately captures essential dimensions of organizational health behavior. This methodological alignment strengthens the reliability and generalizability of the findings. The OACA model assesses eight dimensions of organizational health, namely: strategy, flexibility, governance and supervision, employee welfare, financing and investment, safety and security, productivity, and communication (Tounsi et al., 2021). All OACA items are scored using a 4-point Likert scale, where 1 represents the poorest condition and 4 represents the optimal condition; higher scores thus indicate healthier organizational conditions. For this study, five dimensions from the OACA model were matched with corresponding OHBI dimensions to assess correlations. The analysis revealed strong positive correlations, with Pearson’s *r* coefficients ranging from 0.75 to 0.83, indicating good criterion validity as per Rosenbaum (1989). Detailed results are presented in Tables 15, 16.

**Table 15:** Correlations between dimensions of the OHBI and OH model (OACA).

Items of OHBI and OH model (OACA)	*N*	Pearson r	*p*	Sig.
Awareness, safety, and security	244	0.765**	0.01	0.000
Appreciation and employee welfare	244	0.822**	0.01	0.000
Relation and governance and supervision	244	0.739**	0.01	0.000
Employee engagement and productivity	244	0.752**	0.01	0.000
Internal communication satisfaction and communication	244	0.838**	0.01	0.000

**Correlation is significant at the 0.01 level (one-tailed).

**Table 16:** Final set of 17 items of the five-factor quantitative model of Subscale A of the OHBI.

S. no	Items	Theme
1	I am aware of my organization’s vision, mission, and goals.	Awareness
2	I am implementing to my organization values in my daily work.	Awareness
3	I am aware of my organization external and internal news.	Awareness
4	I am aware of my rights and privileges as an employee in my organization	Awareness
5	The appreciation my line manager shows for my work and accomplishments is satisfactory to me.	Appreciation
6	My company supports my efforts by providing the resources required to accomplish my tasks more effectively.	Appreciation
7	I rarely think about leaving my organization to work someplace else	Appreciation
8	I maintain a very strong relationship with colleagues in other departments.	Relations
9	My relationship with my line manager is perfect.	Relations
10	My family has a good relationship with my organization (they are aware of my work responsibilities, value the services offered by the organization, and stay informed about its news).	Relations
11	I am keen on promoting my organization’s accomplishments and activities, both on my personal social media profiles and during my social engagements.	Engagement
12	During work hours, time flies by without me noticing as I focus on my tasks and responsibilities.	Engagement
13	I am familiar with the professional backgrounds of my colleagues in the same department, their health conditions, and the best ways to interact with them in general.	Engagement
14	I would recommend my organization as a great place to work for my colleagues and family.	Engagement
15	My organization interacts positively with global awareness days, standing out compared to other organizations.	Communication satisfaction
16	I am satisfied with the communication services offered by my organization last year, including email communications, motivational programs, and recreational and interactive activities for employees.	Communication satisfaction
17	Internal communication was able to reach me through various communication channels.	Communication satisfaction

### 4.2 Subscale B: qualitative scale validation

#### 4.2.1 Phase 4

##### 4.2.1.1 Study 6: Subscale B: qualitative item adaptation

In the previous validation research of the OHBI, Subscale B—a qualitative measure—was developed to capture deeper, context-specific elements of organizational health behavior not typically addressed by quantitative metrics. This subscale was designed to elicit narrative insights on three conceptual domains: employee persona, organizational culture, and employee voice, drawing on foundational theories such as the CVF (Cameron and Quinn, 2006), social identity theory (Ashforth and Mael, 1989), and Morrison’s (2014) conceptualization of employee voice.

The earlier validation established the content validity, thematic coverage, and cultural sensitivity of the 10-item, 3-factor qualitative structure through expert review and sector-specific piloting. For the present study, the same validated item framework was retained, but minor revisions were made to terminology and response options to improve clarity, sectoral relevance, and alignment with the workplace context. These adjustments included simplifying phrasing for ease of comprehension across a diverse employee population, updating terminology to reflect current organizational and technological trends and modifying options to ensure organizational culture compatibility that can fit in all sectors.

##### 4.2.1.2 Study 7: cognitive pretesting and face validity

To evaluate the adapted items, a pilot study was conducted with 40 professionals from four key sectors—healthcare, oil and gas, agriculture, and public administration—selected to represent varied organizational environments in Saudi Arabia. This stage focused on assessing face validity, sectoral relevance, and item interpretability. Participants reviewed each item for clarity, comprehension, and contextual appropriateness. Structured cognitive interviews and annotated response forms were used to gather detailed feedback, following Willis (2005) cognitive pretesting guidelines. The findings indicated that respondents considered the items relevant and the language relatable to their work contexts. Minor rewording was performed to enhance professional tone and terminological accuracy. This process confirmed that Subscale B is both theoretically grounded and practically adaptable, enhancing the validity and generalizability of the OHBI in heterogeneous organizational settings (DeVellis, 2016; Boateng et al., 2018). These refinements support the scale’s readiness for integration into future large-scale validation studies.

##### 4.2.1.3 Study 8: interrater reliability assessment

To enhance methodological quality and ensure the reliability of the OHBI qualitative subscale, interrater reliability was assessed for the narrative-based, open-ended responses. Employee responses were collected through the 10-item Subscale B, which included both multiple-choice questions (e.g., preferences for communication channels, appreciation methods, or organizational culture descriptors) and two open-ended questions designed to elicit qualitative feedback on positive and negative aspects of the work environment. Three independent raters, all experts in human resource management and organizational culture, were engaged to code the responses. For the multiple-choice items, raters verified consistency in coding across categorical options, while for the open-ended items, a structured thematic framework was applied. The coding scheme was created using a hybrid approach that combined deductive themes derived from established models (e.g., Schein, 2010; Morrison, 2014) with inductive insights derived from the data. The degree of agreement between raters was quantified using Cohen’s Kappa statistic, which is widely recognized as a robust measure of interrater reliability in qualitative research. The mean Kappa coefficient across all coded items was 0.84, indicating a high level of agreement and confirming the consistency and dependability of the thematic coding (McHugh, 2012). Codes were then transformed into candidate survey items. To ensure conceptual clarity, items were retained only if (a) both coders independently agreed on their relevance, (b) the theme was mentioned by at least 20% of participants (frequency threshold), and (c) the item aligned with the study’s theoretical framework. This process yielded an initial pool of 28 candidate items, which were later reduced to 17 through expert review and factor analysis.

#### 4.2.2 Theoretical foundation and diagnostic contribution

The inclusion of Subscale B enriches the OHBI from a traditional quantitative measurement tool to a multidimensional diagnostic framework. By incorporating qualitative employee insights, the tool captures both observable organizational practices—consistent with Hofstede’s cultural onion model as well as underlying assumptions, as theorized by Schein (2010). The finalized Subscale B consists of 10 items across three core dimensions (see Table 17) and is designed to support both thematic analysis and structured reporting, while maintaining flexibility for diverse organizational and cultural contexts (Table 18).

**Table 17:** Interrater reliability assessment using Cohen’s Kappa.

Rater pair	Number of items coded	Cohen’s Kappa (κ)	Agreement level (McHugh, 2012)
Rater 1 and Rater 2	10	0.83	Almost Perfect
Rater 1 and Rater 3	10	0.86	Almost Perfect
Rater 2 and Rater 3	10	0.82	Almost Perfect
Average	–	0.84	High Reliability

Agreement level based on McHugh’s (2012) interpretation scale.

**Table 18:** The final set of 10 items of the three-factor qualitative method of Subscale B of the OHBI.

S. no	Items	Theme
1	In your opinion, what priorities does your organization focus on the most? A. Employees B. Projects or Customers C. Products or Innovation D. Quality and System	Culture
2	Choose the most appropriate behavior that describes your line manager: A. Supportive—Mentor B. Inspiring—Risktaker C. Productive—Hardworking D. Cautious—Adheres to rules	Culture
3	Choose the most appropriate behavior that describes your colleagues: A. Supportive B. Productive C. Creative D. Cautious—Rule-followers	Culture
4	Identify the top three personal interests that appeal to you: A. Sports and Health B. Technology and Gaming C. Entertainment and Shopping D. Finance and Business E. Restaurants and Cafés F. Education and Development G. Volunteering and Community Service H. Travel and Tourism I. Wildlife and Nature J. Culture and Arts	Persona
5	What are the key aspects you know about your colleagues: A. Their health condition B. Their hobbies and talents C. Their professional and academic history D. How to deal with them F. General information about them E. Not interested	Persona
6	The top four ways that represent appreciation to you are: A. Administrative awards B. Financial compensation C. Highlighting and showcasing my achievements D. Hearing my opinions and including me in key decisions E. Focusing on my mental well-being F. Investing in my professional growth and career G. Verbal appreciation with expressions of thanks H. Material gifts I. Clear information about my rights and benefits J. Financial perks	Persona
7	Best three communication channels to deliver messages for you: A. E-mail B. Intranet/Internal Portal C. Snapchat D. Display Screens E. Telegram F. Text Messages (SMS) G. Whatsapp H. Wall stickers I. Workshops	Persona
8	To whom do you feel the greatest sense of appreciation? A. Your Colleagues B. Your manager C. Your organization	Persona
9	In your opinion, what are the key aspects of our work environment that need improvement and development?	Open-ended
10	In your opinion, what are the key positive aspects of our work environment that we should strive to reinforce?	Open-ended

### 4.3 Scoring for organizational health behavior index

#### 4.3.1 Scoring for Subscale A (quantitative scale)

The final 17-item subscale was developed from this study to measure Organizational Health and Behaviour. The scale asks respondents to rate each item on a 5-point Likert scale ranging from 1 (strongly disagree) to 5 (strongly agree). There are no reverse-scored items in the scale. Subscale A is further divided into five dimensions: awareness (four items), appreciation (three items), relations (three items), employee engagement (four items), and internal communication satisfaction (three items). The scores on each dimension are obtained by adding all the items of their respective dimensions. A total score of a dimension is obtained by adding responses to all the items of that dimension. Similarly, the score on Subscale A is obtained by adding scores/responses of all 17 items and can range from a minimum score of 17 to a maximum score of 85. A normalized score higher than the normalized mean score is an index of good organizational health and behavior, and a lower score is an index of poor organizational health and behavior (see Table 15).

#### 4.3.2 Scoring of Subscale B (qualitative scale)

The scoring of the three dimensions (employee persona, organizational culture, and employee voice) of Subscale B is undertaken in different ways.

Scoring for Employee Persona: Employees’ persona is evaluated on five items based on five themes: their interests, their level/type of engagement, their communication method, their definitions of appreciation, and their level of loyalty. There is no calculation of a numerical score in this scoring process. Instead, for each factor, qualitative data are obtained and assessed. Multiple-choice questions are used to obtain qualitative data. Content analysis is part of the scoring procedure.

Scoring for Organizational Culture: Three items, each with four multiple-choice answers that represent several cultural kinds (clan culture, market culture, adhocracy, and hierarchy culture), are evaluated as part of the scoring process for the aspects of organizational culture. Whether the culture is created by the organization, leaders, or employees will be shown by the conclusion taken from the responses to the three questions. The scoring process can be put into practice as follows. Four multiple-choice options (A, B, C, and D) reflecting several cultural kinds (clan, market, adhocracy, and hierarchy) should be presented to responders. For this choice, respondents are asked to select the response that most accurately reflects how they see the culture of the company.

Scoring for Employee Voice: This dimension consists of two open-ended items, and the scoring is based on thematic analysis. There are no calculations for this dimension. Opinions are gathered to hear the employee’s voice. The voices lead to either a positive or negative theme.

## 5 Discussion

The purpose of this study was to construct and validate the OHBI as a multi-dimensional diagnostic tool to measure structural and perceptual sides of organizational health from a Saudi Arabian perspective. Consistent with Saudi Arabia’s Vision 2030 to improve human capital and institutional quality (Abubakar et al., 2024), the OHBI encompasses a combined approach of quantitative and qualitative methods, consisting of seven studies across four developmental stages. The instrument provides a full review of the functioning of the organization, internal communication and engagement of employees.

The Organizational Health Behavior Index comprises five factors in the subscale, one of which is quantitative. These dimensions, which can be measured quantitatively, are crucial, as per the literature available. Awareness, for example, refers to employees’ understanding of organizational structures, people, and ongoing activities. It includes both static knowledge (e.g., location of departments) and dynamic information (e.g., team availability and workflow progress), which are essential for informed decision-making and productivity (Khuen et al., 2015). Appreciation—manifested through recognition and rewards—plays a vital role in enhancing employee engagement and performance. Research suggests that organizations with strong recognition programs see higher productivity, revenue, customer retention, and employee satisfaction. A culture of appreciation fosters motivation, encouraging employees to strive for excellence (Mounika et al., 2021). Similarly, positive relations, characterized by meaningful interactions between employees and supervisors, contribute to a strong sense of community and overall workplace morale. High-quality relationships provide employees with essential resources for setting and achieving goals, reinforcing a sense of job satisfaction (Kumar and Manjula, 2017; De Massis, 2018). Referring to the EST theory, this study predicts that employee relations as part of a well-being working environment will lead to an increased sense of job satisfaction (Bulińska-Stangrecka and Bagieńska, 2021). Employee engagement, defined as the emotional commitment to organizational goals, remains a critical factor for achieving sustainable performance (Abdelwahed and Doghan, 2023; Shrestha, 2019). Finally, communication satisfaction —the quality and effectiveness of internal communication—serves as a linchpin in fostering transparency, psychological safety, and employee well-being. These fundamentals of a healthy work environment cannot be achieved without effective communication, given that it has a major role in alleviating tensions and mental health issues (Anwar and Abdullah, 2021).

The quantitative validation process for Subscale A was rigorous and theory-driven. Initial item generation and refinement, grounded in existing literature and expert input, ensured a strong foundation for construct representation. The results of the EFA showed a five-factor structure—awareness, appreciation, relations, employee engagement, and communication satisfaction —that was both stable and accounted for more than 63% of the total variance. The high factor loading and no large cross-loadings suggests good construct clarity and item convergence. These results are in accordance with the organizational behavior literature that highlights that antecedents of engagement and health are multidimensional (Bakker and Demerouti, 2007; Kelloway and Barling, 2010). CFA on a large national sample (*n* = 7,548) yielded excellent model fit indices (CFI = 0.978, RMSEA = 0.041), further substantiating the factorial structure of the OHBI-17. All items loaded significantly onto their respective latent constructs, with most standardized loadings exceeding 0.75. By conducting CFA on an independent large-scale sample (*n* = 7,548), this study goes beyond exploratory analyses and provides strong support for the construct validity of the OHBI. This step addresses a limitation common in organizational health research, where many indices rely solely on Cronbach’s alpha or EFA without confirmatory validation. High factor loadings for indicators such as EE3 (0.871) and CS2 (0.871) suggest particularly salient expressions of engagement and communication within the organizational context. Reliability coefficients for all subscales surpassed the 0.85 threshold, supporting the internal consistency of the OHBI.

Convergent and discriminant validity were confirmed through AVE, CR, and Fornell-Larcker criterion, and HTMT ratios. The AVE varied from 0.565 to 0.663, and the CR from 0.830 to 0.869, which showed good convergent validity. The low between-construct correlation and HTMT values, which are clearly under 0.85, also indicate that the five dimensions of this scale are theoretically related but different, and suggest a successful operationalization (Fornell and Larcker, 1981; Henseler et al., 2015). Second, criterion validity was established by significant correlations between OHBI scores and a predetermined valid Organizational Health Model (OACA). This is evidenced by the range of Pearson’s *r* values observed (0.739–0.838), indicating a strong alignment of OHBI dimensions with established constructs such as safety, employee well-being, productivity, and communication. This provides evidence that the OHBI measures an important aspect of organizations and can be used as a valid diagnostic tool in practice.

While quantitative indicators provide structured statistical insights into organizational health, they may overlook subtle, context-specific, and deeply embedded aspects of organizational culture, employee experiences, and behavioral norms. Subscale B addresses this gap by integrating a qualitative dimension into the OHBI framework, thereby enabling a richer, narrative-based understanding of organizational health and behavior.

Drawing on established theories—including the CVF (Cameron and Quinn, 2006), social identity theory (Ashforth and Mael, 1989), and the employee voice literature (Morrison, 2014)—Subscale B captures three interrelated domains: organizational culture, employee persona, and employee voice. These domains illuminate how shared norms, leadership styles, interpersonal relationships, and employee identity influence engagement, trust, and workplace dynamics. For instance, strong organizational cultures characterized by fairness, trust, and transparency (Rasooly Kalamaki et al., 2020) have been linked to enhanced participation in decision-making and improved organizational health. Similarly, respecting employee interests and systematically incorporating feedback can foster innovation, morale, and retention.

The qualitative items in Subscale B were adapted from the previously validated OHBI framework, with minor revisions to terminology and response options to improve clarity, sectoral relevance, and cultural alignment for use across diverse industries in Saudi Arabia. Cognitive pretesting with 40 professionals from four key sectors—healthcare, oil and gas, agriculture, and public administration—confirmed the face validity, contextual relevance, and linguistic appropriateness of the items (Willis, 2005). Minor rewording ensured a professional tone and alignment with contemporary workplace practices. Open-ended responses were subjected to interrater reliability assessment using three expert raters in HR and organizational culture. A hybrid coding scheme combining deductive themes from Schein (2010) and Morrison (2014) with inductive insights from the data yielded a mean Cohen’s Kappa of 0.84, indicating almost perfect agreement (McHugh, 2012) and reinforcing the methodological rigor of the qualitative analysis.

By integrating this dimension, the OHBI evolves from a purely quantitative measure into a multidimensional diagnostic framework. Subscale B equips organizations with actionable insights into intangible cultural indicators and employees’ affective orientations—elements often overlooked in numerical diagnostics—thereby enhancing its applicability for performance improvement, cultural transformation, and strategic HR planning.

### 5.1 Theoretical and practical contributions

The OHBI advances both theoretical and applied understandings of organizational health in several ways. First, the integration of employee engagement, communication satisfaction, and relational health into a cohesive framework reflects contemporary models of thriving organizations (Albrecht et al., 2015; De Smet et al., 2023). Second, the multidimensional approach accommodates both observable practices and subjective interpretations, bridging the epistemological gap between objectivist and constructivist paradigms in organizational assessment.

Higher OHBI ratings are often linked to greater workforce engagement. The index can act as a strategic guide for fostering a supportive work environment that enhances employee satisfaction, productivity, and retention. Additionally, organizations can use the OHBI to compare their health behavior scores against industry benchmarks or best practices, gaining insights into their relative standing and effective strategies for improvement. This comparative analysis helps shape strategic decision-making, allowing organizations to align their goals and initiatives with the key factors influencing overall organizational health.

Furthermore, the OHBI can serve as a performance measurement tool, promoting a culture of continuous improvement. Recognizing and rewarding teams or departments that contribute positively to organizational health behavior encourages sustained progress. It can also track the impact of major organizational changes on health behavior and support ongoing learning and development efforts. Regular evaluations provide insights into the effectiveness of past initiatives and help refine future strategies.

Moreover, organizations can utilize the OHBI to assess leadership’s impact on organizational health behavior. It helps identify areas where leadership development and training may be necessary to cultivate a more positive workplace culture.

Practically, the OHBI offers a validated, sector-neutral tool suitable for public, private, and semi-government organizations. Its scalability, high internal consistency, and psychometric integrity make it particularly valuable for benchmarking, strategic planning, and HR interventions in alignment with Saudi Arabia’s Vision 2030. The inclusion of qualitative indicators enhances diagnostic sensitivity by uncovering deep-seated cultural values and employee sentiments that traditional surveys may miss.

### 5.2 Strengths, limitations, and future research

The study’s strengths lie in its large and diverse sample, multi-phase design, and rigorous validation procedures. The dual-scale structure addresses both surface-level indicators and deeper organizational dynamics, providing a comprehensive view of health behaviors. However, several limitations warrant attention. First, although the sample is large, the use of non-probability sampling may limit generalizability beyond Saudi organizational contexts. Second, while Subscale B’s qualitative insights are rich, they require trained coders for thematic analysis, which may constrain widespread adoption in resource-constrained settings.

Future research should extend the validation of OHBI to international and cross-cultural contexts to test its universality and cultural adaptability. Moreover, longitudinal studies could explore the OHBI’s predictive validity in relation to organizational outcomes, such as performance, retention, and innovation. Finally, integrating the OHBI into organizational development programs can provide feedback loops for continuous improvement and cultural transformation.

## 6 Conclusion

In summary, this study successfully developed and validated the OHBI, demonstrating its reliability and strong factorial structure. The findings confirm that OHBI is a robust tool for assessing key aspects of organizational health, making it valuable for both researchers and practitioners. By leveraging this index, organizations can gain meaningful insights into workplace dynamics, employee well-being, and engagement, ultimately fostering a healthier and more productive work environment. The OHBI serves as a valuable tool for organizations to identify areas requiring improvement. By analyzing ratings across various organizational health behavior dimensions, managers can pinpoint priority areas and allocate resources accordingly. A thorough assessment of organizational health behavior enables targeted interventions to address specific weaknesses. Organizations can implement specialized training programs, workshops, or initiatives to enhance areas with lower scores.

In summary, the OHBI provides valuable insights into an organization’s overall health behavior, highlighting specific areas for enhancement. By leveraging this tool, businesses can implement targeted interventions, improve employee engagement, and align their strategies with key health behavior factors, ultimately fostering a more productive and sustainable work environment.

## Data Availability

The original contributions presented in the study are included in the article/Supplementary material, further inquiries can be directed to the corresponding author.
