# Correction: Development and validation of the organizational health behavior index: a mixed-methods instrument for measuring organizational health

**DOI:** 10.3389/fpsyg.2026.1923122

**Published:** 2026-07-28

**Authors:** Abad Alzuman, Muath Jaafari, Zaiba Ali, Rahila Ali, Lina Ibrahim Bakadam

**Affiliations:** 1 Department of Management, College of Business Administration, Princess Nourah Bint Abdulrahman University, Riyadh, Saudi Arabia; 2 Organizational Culture and Internal Communication, ICM, Amjad Watan, Riyadh, Saudi Arabia.; 3 Faculty of Management, Jagran Lakecity University, Bhopal, India

**Keywords:** organizational health, awareness, appreciation, relations, engagement, communication satisfaction, organizational culture, employee persona

In the published article an incorrect **Funding** statement was provided as “This study was supported by internal research funding from Princess Norah University, Riyadh, SA, which provided institutional support for data collection and analysis.”

The correct **Funding** statement reads:

The author(s) declared that financial support was received for this work and/or its publication. The authors extend their appreciation to the Deanship of Scientific Research and Libraries in Princess Nourah Bint Abdulrahman University for funding this research work through the Research Group project, Grant No. (RG-1445-0070).

In addition, affiliation Department of Management, College of Business Administration, Princess Nourah Bint Abdulrahman University, Riyadh, Saudi Arabia was erroneously given as Management Department, College of Business Administration, Princess Nourah Bint Abdulrahman University, Riyadh, Saudi Arabia.

Author Zaiba Ali was erroneously assigned to affiliation Organizational Culture and Internal Communication, ICM, Amjad Watan, Riyadh, Saudi Arabia. The correct affiliation for author Zaiba Ali is Department of Management, College of Business Administration, Princess Nourah Bint Abdulrahman University, Riyadh, Saudi Arabia.

Author Rahila Ali was erroneously assigned to affiliation Management Department, College of Business Administration, Princess Nourah Bint Abdulrahman University, Riyadh, Saudi Arabia The correct affiliation for author Rahila Ali is Faculty of Management, Jagran Lakecity University, Bhopal, India.

Author Lina Ibrahim Bakadam was erroneously assigned to Faculty of Management, Jagran Lakecity University, Bhopal, India. The correct affiliation for author Lina Ibrahim Bakadam is Department of Management, College of Business Administration, Princess Nourah Bint Abdulrahman University, Riyadh, Saudi Arabia.

The original version of this article has been updated.

