# Correction: Development and validation of the organizational health behavior index: a mixed-methods instrument for measuring organizational health

**DOI:** 10.3389/fpsyg.2026.1970038

**Published:** 2026-09-01

**Authors:** Abad Alzuman, Muath Al Jaafari, Zaiba Ali, Rahila Ali, Lina Ibrahim Bakadam

**Affiliations:** 1 Department of Management, College of Business Administration, Princess Nourah Bint Abdulrahman University, Riyadh, Saudi Arabia; 2 Organizational Culture and Internal Communication, ICM, Amjad Watan, Riyadh, Saudi Arabia; 3 Faculty of Management, Jagran Lakecity University, Bhopal, India

**Keywords:** organizational health, awareness, appreciation, relations, engagement, communication satisfaction, organizational culture, employee persona

In the published article, affiliation Organizational Culture and Internal Communication, ICM, Amjad Watan, Riyadh, Saudi Arabia was incorrectly assigned to author Dr Zaiba Ali.

The correct affiliation for author Dr Zaiba Ali is: Department of Management, College of Business Administration, Princess Nourah Bint Abdulrahman University, Riyadh, Saudi Arabia.

The original version of this article has been updated.

